# An engineered intradermal microneedle-array device enhances the cellular immune responses in a guinea pig model

**DOI:** 10.3389/fimmu.2026.1828400

**Published:** 2026-05-19

**Authors:** Marie-Edith Nepveu-Traversy, George (Giorgi) Babuadze, Darlene Potterton, Marc-Antoine de La Vega, Ales Alajbegovic, Jacinthe Julien, Andrew Winterborn, Gary Kobinger

**Affiliations:** 1 Global Urgent and Advanced Research and Development, Batiscan, QC, Canada; 2 University of Texas Medical Branch, Galveston, TX, United States; 3 Queen’s University, Kingston, ON, Canada; 4 InocuJect Corporation, Ottawa, ON, Canada; 5 Centre de Recherche en Sciences Animales de Deschambault (CRSAD), Deschambault, QC, Canada

**Keywords:** cellular immune response, DNA vaccine, electroporation, humoral immune response, intradermal vaccination, neutralizing antibodies

## Abstract

**Introduction:**

Intradermal (ID) vaccination can be highly immunogenic due to the density of antigen-presenting cells in the skin, but reliable ID delivery remains technically challenging. We evaluated a programmable microneedle-array intradermal injector, InocuJect, designed to distribute vaccine micro-deposits across a defined dermal area.

**Methods:**

In female Hartley guinea pigs, we compared vaccination with the automated ID device to conventional Mantoux intradermal injection followed by electroporation (ID+EP) using two vaccine platforms: a DNA vaccine encoding SARS-CoV-2 spike (pIDV-II-SARS-CoV-2) and the licensed virus-like particle vaccine GARDASIL®9.

**Results:**

For the DNA vaccine, both methods induced spike-specific and pseudovirus-neutralizing responses by day 56, with similar neutralizing titers between groups. For GARDASIL®9, both delivery methods generated comparable HPV16 L1-specific IgG at the final time points. In contrast, the automated ID device induced stronger antigen-specific cellular responses, as reflected by increased IFN-γ RNA and protein levels following peptide stimulation.

**Conclusions:**

These findings suggest that spatially distributed dermal delivery using a microneedle-array-based intradermal injector is associated with enhanced IFN-γ-based cellular immune readouts, while maintaining robust humoral responses across distinct vaccine platforms.

## 1 Introduction

The route of vaccine administration is a key determinant of immunization efficacy. Among the available methods, intradermal (ID) vaccination has demonstrated remarkable immunogenicity due to the skin’s rich network of immune cells, including antigen-presenting cells such as Langerhans cells, dermal dendritic cells, and macrophages, as well as skin-resident memory T lymphocytes that constitute an effective first line of immune defense and contribute to vaccine-specific adaptive immunity (1–3). Despite these advantages, most vaccines are still administered intramuscularly because this route is technically simple, convenient, and requires minimal training. However, numerous studies have shown that intradermal delivery can elicit stronger immune responses than intramuscular injection (4–6), while also reducing antigen dose requirements—a strategy successfully implemented during the 2022 mpox outbreak, when the U.S. Food and Drug Administration (FDA) authorized intradermal administration to extend vaccine supplies (7). Moreover, intradermal vaccination can minimize or eliminate the need for adjuvants, which are often associated with local and systemic side effects (8).

Despite its immunological advantages, the widespread adoption of intradermal vaccination has been limited by two main factors: the small injection volume capacity of the dermis and the technical expertise required for precise Mantoux-style injections. To overcome these limitations, alternative skin-targeted delivery technologies such as microneedle arrays have been developed. Microneedle-array systems are broadly used to describe minimally invasive intradermal delivery platforms designed to deposit antigens within the epidermis and superficial dermis. While definitions may vary across the literature, these systems are primarily characterized by their ability to bypass the stratum corneum and achieve controlled delivery into the dermal immune microenvironment, typically at depths on the order of ~1 mm. This functional definition, rather than strict geometric criteria, is widely adopted in the field of skin-based vaccination (9–11).

For example, microneedle patches are designed to deposit vaccine antigens reproducibly within epidermal and dermal layers rich in antigen-presenting cells, and numerous preclinical and clinical studies demonstrate robust humoral and cellular responses, often comparable or superior to conventional intramuscular injection and with dose-sparing potential. In addition to simplifying administration and reducing dependence on technically demanding intradermal injections, microneedle patches can enhance immune activation by directly accessing dense networks of skin dendritic cells. Nevertheless, limitations such as restricted antigen payload per patch, formulation and loading constraints for nucleic-acid vaccines, and persistent barriers to automated, large-scale aseptic manufacturing remain important considerations for widespread implementation (12–14). To address these challenges, our team previously developed and validated a prototype intradermal delivery device designed to improve DNA vaccine performance. This prototype demonstrated promising results in guinea pigs, rabbits and rhesus macaques, inducing stronger T- and B-cell responses than traditional intramuscular or intradermal injections, and generating higher antibody titers compared to DNA tattooing (15). However, its practical application was limited by its complexity and the need for extensive operator training, restricting its broader use.

Another method known for its high efficiency in DNA vaccine delivery is the use of electroporation (EP). However, it presents several limitations that restrict its routine use in both preclinical and clinical settings. EP requires specialized equipment, trained operators, and careful control of electrical parameters and electrode configuration to maximize DNA uptake while minimizing pain and tissue damage (16, 17). The localized electrical pulses induce transient but marked tissue damage and inflammation that enhance immunogenicity but also affect tolerability and make immune readouts highly dependent on the chosen pulse parameters (16, 18). Immune responses after EP are sensitive to pulse patterns, voltage–current settings, and electrode geometry, such that suboptimal or non-standardized conditions can increase variability in antigen expression and immune responses across animals (16, 19). In addition, the geometry and contact area of EP electrodes constrain the effective electric field and DNA delivery to a relatively small region of epidermis and dermis under the applicator, limiting the treated area available for interaction with skin-resident antigen-presenting cells (19, 20). These constraints highlight the need for alternative intradermal delivery technologies capable of providing high efficiency, wider dermal coverage, and more controlled delivery without the procedural complexity associated with EP.

Taking these limitations into account and building on prior work with a prototype intradermal delivery device, we developed InocuJect, an automated intradermal vaccine delivery system. The device is designed to distribute vaccine deposits in a controlled manner across the dermis while simplifying the injection procedure compared to electroporation. By promoting spatially distributed dermal deposition across a 2.5 × 2.5 cm area, this approach increases the likelihood of antigen encounter with a larger number of skin-resident antigen-presenting cells. By depositing multiple micro-volumes of vaccine across a broader dermal surface, the device enables access to epidermal Langerhans cells, dermal dendritic cells, and skin-resident T lymphocytes, which may collectively contribute to enhanced immune activation compared to conventional Mantoux injection alone or when combined with electroporation (21, 22). The programmable penetration depth and controlled dwell time are designed to place antigen within the dermis, which is rich in immune cell populations, and may contribute to more consistent intradermal deposition across injections and animals. Similar approaches using microneedle-based systems with defined needle length have been reported to improve spatial control of antigen delivery (23). Spatially distributed intradermal antigen delivery has been associated in previous microneedle-array vaccination studies with efficient germinal-center and T-cell responses (22, 24). In contrast, electroporation-based intradermal delivery is typically characterized by highly localized delivery and, transient local inflammation driven by electrical field application. This contextual framework informed the comparative evaluation of the automated ID device in the present study.

The objective of this study was to evaluate the immunogenic performance of the automated ID device in a guinea pig model using two types of vaccines: a DNA vaccine (pIDV-II-SARS-CoV-2) previously validated with our first prototype and a commercially available VLP-based vaccine (GARDASIL^®^9). The efficacy of the automated ID device was compared to intradermal injection followed by electroporation, a method known for its high efficiency in DNA vaccine delivery (19, 25).

## 2 Materials and methods

### 2.1 Animals

Female Hartley guinea pigs (6–8 weeks old, ~250–300g) were purchased from Charles River. Only female animals were used to reduce sex-related variability in immune responses and to minimize the number of animals required for this study. All animals were housed and handled in accordance with Canadian Council on Animal Care guidelines, and all procedures were approved by the Animal Care Committee of CRSAD and the Queen’s University Animal Care Committee (UACC) under protocol numbers 2021-CDI-443 and 2021-2145, respectively. For euthanasia, guinea pigs were anesthetized with 1.5–4% isoflurane and then euthanized by cardiac exsanguination followed by decapitation. Of note, one animal in the intradermal followed by electroporation group was lost due to a bacterial infection unrelated to vaccination; data up to week 4 were retained for analysis.

### 2.2 Vaccines

The first vaccine is a DNA-based vaccine, pIDV-II-SARS-CoV-2 spike, previously used in similar experiments with the first intradermal prototype device, as described elsewhere (15). Briefly, a consensus sequence of the SARS-CoV-2 spike protein was generated by aligning 92 spike sequences retrieved from NCBI GenBank and subsequently codon-optimized for human expression. The resulting gene fragment was synthesized and cloned into the pIDV-II plasmid—a modified pVAX backbone containing a Neo/Kanamycin resistance gene for selection in *E. coli*, and a hybrid CMV–chicken β-actin (CAG) promoter to drive robust expression across diverse mammalian cell types. The construct also includes the Woodchuck Hepatitis Virus post-transcriptional regulatory element (WPRE) to enhance post-transcriptional regulation of gene expression (15). The vaccine was produced at a high concentration (2.5 mg/ml) in saline (Aldevron, ND, USA).

The commercially available 9-valent Human Papillomavirus (HPV) vaccine, Gardasil^®^9 (Merck), was used. This 0.5mL suspension contains virus-like particles (VLPs) from nine HPV types, including 60µg of HPV16 L1, with additional VLPs from HPV6, 11, 18, 31, 33, 45, 52, and 58 (26).

### 2.3 Device

The InocuJect automated intradermal microneedle-array-based injector was used for vaccine administration (US Patent Application US20240075215A1, 2024). The device delivers liquid formulations into the skin via repeated insertions of a multi-needle array composed of fine-gauge needles, with user-defined penetration depth, cycle timing, and controlled spatial repositioning across the application area. The needle array is arranged in a fixed geometric configuration that enables consistent spatial coverage through repeated micro-injections across the targeted dermal surface. For this study, parameters were set to target dermal delivery in guinea pig skin (nominal penetration depth: 1 mm) without subcutaneous deposition. A 10-needle array fitted with 33-gauge needles was used, with a total delivered volume of 0.5 mL per animal. The device utilizes fine-gauge needles with dimensions slightly larger than those used in some hollow microneedle systems; however, due to their shallow penetration depth and intradermal targeting, the system is functionally aligned with microneedle-array based delivery approaches. Before each immunization session, the device was calibrated to confirm volumetric delivery accuracy by dispensing saline and verifying mass. Delivery settings were kept identical across all animals within each experiment. No device malfunctions, needle clogging, or loss of pressure were observed during the study.

### 2.4 Immunization of guinea pigs

First, a pilot study was conducted to determine the optimal dosage of the commercial Gardasil^®^9 vaccine in guinea pigs. Three guinea pigs per group were injected intramuscularly (IM) with 0.2 mL of four different dilutions of the vaccine prepared in sodium chloride injection USP 0.9% (1/25, 1/50, 1/100, and 1/200). Serum IgG responses against HPV16 L1 were measured over four weeks, and the 1/100 dilution was chosen for subsequent experiments as it elicited strong but non-saturating antibody levels suitable for comparative immunogenicity analysis.

The main study consisted of four groups, each comprising six guinea pigs, which received one of two vaccines: Gardasil^®^9 or pIDV-II-SARS-CoV-2 (250 µg). Each vaccine was administered using two distinct delivery methods: (1) the automated ID device delivering 0.5 mL per guinea pig, and (2) intradermal Mantoux injection, performed using a 1 mL tuberculin syringe fitted with a 27-gauge, ½-inch needle (0.1 mL), followed by electroporation with the CELLECTRA 3P ID array (Inovio, California, USA) applying a current of 0.2 A and a 4-second delay between electrical pulses. A booster dose was administered four weeks after the prime injection using the same conditions. Serum samples were collected prior to the first injection and on days 28, 42, and 56. Spleens were harvested at day 56 upon animal sacrifice to allow isolation of splenocytes.

The total injection volume differed between groups due to inherent characteristics of the delivery methods rather than differences in antigen dose. The automated ID device is designed to distribute vaccine micro-deposits across a 2.5 × 2.5 cm dermal area, which requires a larger total volume (0.5 mL) to ensure uniform dermal coverage. In contrast, the Mantoux technique is limited to a single 0.1 mL bolus, which represents the maximal volume that can be safely retained in a single intradermal bleb without leakage. Importantly, the antigen dose delivered in both groups was identical, and prior intradermal vaccination studies have demonstrated that antigen quantity, but not injection volume, is the primary determinant of humoral and cellular immunogenicity in small animal models (5). Thus, the difference in volume reflects delivery mechanics rather than dose or immunogenic potential.

Local dermal tolerability was assessed at the intradermal vaccination site immediately after administration. For animals vaccinated using the automated ID device, evaluations were performed at the application area contacted by the microneedle array. For the ID + EP group, assessments were conducted at both the Mantoux injection site and the electroporation electrode contact area. Injection sites were examined for erythema, edema, bleeding, wheal persistence, crust formation, and ulceration. Local reactions were graded qualitatively by trained personnel using standardized observational criteria. General animal well-being, including grooming behavior, feeding patterns, and activity levels, was also monitored throughout the study period.

### 2.5 ELISA to evaluate specific IgG

To assess the specific IgG response to Gardasil^®^9, recombinant HPV type 16 major capsid L1 virus-like particles (VLPs) (DAGF-229, Creative Diagnostics, NY, USA) were used to coat 96-well clear high-binding plates (767071, Greiner Bio-One) at 50 ng/well. Although GARDASIL^®^9 contains VLPs from nine HPV types, HPV16 L1 was selected as the antigenic readout for humoral and cellular assays because it is the immunodominant component responsible for the majority of vaccine-induced neutralizing activity and is widely used as a surrogate marker of multivalent HPV vaccine immunogenicity. Previous studies have demonstrated strong cross-correlation between HPV16 L1 specific IgG and the broader antibody response induced by GARDASIL^®^9 across animal models and humans, making it a validated and sensitive biomarker for comparative immunogenicity assessments (27). Focusing the analysis on HPV16 L1 thus enables robust quantification while avoiding signal saturation associated with polyvalent antigen mixtures. To evaluate the SARS-CoV-2 spike-specific IgG response, recombinant full-length spike protein produced and purified from *E. coli* (Université Laval, Quebec, Canada) was similarly coated at 50 ng/well in 96-well plates. All plates were blocked with 200 µL of phosphate-buffered saline (PBS) containing 0.1% Tween-20 and 3% milk for 2 hours at room temperature (RT). Serum samples were diluted 1:100 in PBS-T and incubated for 2 hours at RT. Plates were then washed three times with 200 µL PBS-T, with shaking between washes, using a microplate washer (Biochrom Anthos Fluido 2, USA).

Secondary goat anti–guinea pig IgG H&L (HRP) antibody (ab6908, Abcam) was added at a 1:5000 dilution and incubated for 1 hour at RT. After three additional washes with PBS-T, 75 µL of 3,3’,5,5’-tetramethylbenzidine (TMB) substrate (002023, Invitrogen, USA) was added to each well to develop the signal. The reaction was stopped with 75 µL of 0.5 N sulfuric acid, and absorbance was measured at 450 nm using a microplate reader (MBI Evolution Microplate Reader, Montreal, Canada).

### 2.6 Isolation and stimulation of splenocytes

Spleens from guinea pigs injected with Gardasil^®^9 were collected on day 56 in PBS containing 2% FBS and processed for splenocyte isolation as follows. Spleens were homogenized using a 70 µm cell strainer (Corning, 352350) and resuspended in PBS. Splenocytes were isolated using Lymphoprep™ (Stemcell, 07851) in SepMate™-15 tubes (Stemcell, 15415) by centrifugation at 3,000 rpm for 20 minutes at room temperature (with the brake off). Cells at the interphase were collected and washed twice with PBS. Splenocytes were then resuspended in FBS containing 10% dimethyl sulfoxide (DMSO) (D8418, Millipore Sigma, USA) and stored in liquid nitrogen until use.

Splenocytes were thawed in complete RPMI medium (10-040-CV, Corning) supplemented with 10% FBS (080450, Multicell Wisent) and penicillin–streptomycin (14140-122, Gibco). Viable cells were counted using 0.4% Trypan Blue (EBT-001, NanoEntek, Korea) and the EVE automated cell counter (NanoEntek, Korea). A total of 300,000 viable cells were seeded in 48-well plates in 200 µL of complete RPMI and allowed to rest for 4 hours at 37 °C in a 5% CO_2_ atmosphere before stimulation. For antigen-specific stimulation, cells were incubated with a pool of 124 HPV16 L1-specific peptides (PepMix Q9WLQ6, JPT Peptides, USA) at a final concentration of 1 µg/mL. Concanavalin A (Con A) (8 µg/mL; 00-4978-93, Invitrogen) was used as a positive control, and 0.5% DMSO was included to confirm stimulation specificity. Non-stimulated cells served as negative controls.

### 2.7 Total RNA isolation and RT-qPCR

Total RNA from splenocytes was extracted 16 hours post-stimulation using the GeneJET™ RNA Purification Kit (K0731, Thermo Fisher Scientific, USA), following the manufacturer’s protocol. Briefly, cells were pelleted at 250 × g for 5 minutes and washed with PBS. The cell pellets were lysed in the provided lysis buffer supplemented with β-mercaptoethanol. After ethanol addition, the lysate was transferred to a purification column and centrifuged at 12,000 × g for 1 minute. The column was washed sequentially with wash buffer I and wash buffer II, and RNA was eluted in 50 µL of nuclease-free water. RNA concentration and purity were determined using a NanoDrop ND-1000 spectrophotometer (Thermo Fisher Scientific, USA).

Extracted RNA was treated with DNase I (EN0521, Thermo Scientific, USA) to remove genomic and residual DNA by incubation at 37 °C for 30 minutes. The reaction was stopped by adding 10% 50 mM EDTA, followed by incubation at 65 °C for 10 minutes. DNA-free RNA was used for the detection of interferon gamma (IFN-γ) and RNase P (GenBank accession no. XM_005000167.1, used as a housekeeping gene) by direct RT-qPCR using previously described primers and probes (28, 29) specific for guinea pig. The IFN-γ primers and probe were: forward 5’-CAT GAA CAC CAT CAA GGA ACA AAT-3’, reverse 5’-TTT GAA TCA GGT TTT TGA AAG CC-3’, and probe 5’-/56-FAM-TTC AAA GAC/ZEN/AAC AGC AGC AAC AAG GTG C/3IABkFQ/-3’. The RNase P primers and probe were: forward 5’-GGA TTT AGA CCT AAG AGC G-3’, reverse 5’-GAG CGG CAG TTT CCA CCA TT-3’, and probe 5’-/56-FAM-TTC TGA TCT/ZEN/GAA GGC TTT GCG TG/3IABkFQ/-3’ (Integrated DNA Technologies, USA).

RT-qPCR reactions were carried out in a 10 µL final volume using a LightCycler^®^ 96 Real-Time PCR System (Roche Diagnostics GmbH, Basel, Switzerland). Each reaction contained 2 µL of RNA, 0.1 µL (100 µM) of each primer, 0.25 µL (20 µM) of the probe, 2.5 µL of TaqMan Fast 1-Step RT-PCR Master Mix (4444434, Thermo Fisher Scientific, USA), and 5.05 µL of nuclease-free water (AM9937, Invitrogen, USA).

Thermal cycling conditions were as follows: reverse transcription at 50 °C for 10 minutes, denaturation at 95 °C for 2 minutes, followed by 50 amplification cycles of 95 °C for 10 seconds, 55 °C for 20 seconds, and 68 °C for 10 seconds. Data were analyzed using LightCycler 96 software in the absolute quantification mode, and the cycle number at which fluorescence exceeded the threshold was recorded as the cycle threshold (Ct) value.

### 2.8 ELISA to evaluate IFN-γ protein levels

96-well ELISA plates previously coated overnight with guinea pig IFN-γ capture antibody V-E4 (kindly provided by Dr. Hubert Schäfer) diluted to 20 µg/mL in PBS (50 µL per well) were blocked for 1 hour at 37 °C with PBS-T-Milk 3%. The supernatants from splenocytes stimulated for 48 hours were collected by centrifugation at 500 × g for 5 minutes. Subsequently, 80 µL of each sample supernatant and the positive control consisting of supernatant from mitogen-stimulated guinea pig T lymphocytes in tissue culture medium (kindly provided by Dr. Hubert Schäfer) were added in duplicate to the wells and incubated for 1 hour at 37 °C. Plates were then washed three times with PBS-T, and the biotin-conjugated detection antibody NG-3 (kindly provided by Dr. Hubert Schäfer) diluted 1:500 in PBS-T was added and incubated for 1 hour at RT.

After three additional washes, streptavidin-HRP (557630, BD Biosciences) diluted 1:100 in PBS-FBS 10% (50 µL per well) was added and incubated for 1 hour at RT. Plates were washed three more times and once with PBS before adding 75 µL of TMB substrate (002023, Invitrogen, USA) to develop the signal. The reaction was stopped with 75 µL of 0.5 N sulfuric acid, and absorbance was measured at 450 nm using a microplate reader (MBI Evolution Microplate Reader, Montreal, Canada).

### 2.9 SARS-CoV-2 neutralizing assay

To generate SARS-CoV-2-Δ19-D614G–pseudotyped lentiviruses, 293T/17 cells (ATCC, CRL-11268) cultured in DMEM supplemented with 10% FBS and 1% penicillin–streptomycin at 37 °C in a 5% CO_2_ atmosphere were co-transfected, as previously described with minor modifications (30). Cells were transfected with 3 µg of pcDNA3.1-spike-Δ19-D614G, 3 µg of psPAX2 (gift from Didier Trono, Addgene #12260), and 4 µg of pHAGE-CMV-Luc2-IRES-zsGreen (BEI Resources, NR-52516) using Lipofectamine 2000 (11668, Invitrogen, USA). Supernatants were harvested 48 hours post-transfection and filtered through a 0.45 µm membrane. The specificity of the assay was validated prior to testing serum samples using commercially available neutralizing antibodies (BEI Resources, NIAID, NIH: Monoclonal Anti–SARS-CoV-2 Spike Glycoprotein RBD–mFc Fusion Protein, produced *in vitro*, NR-53795) and negative control sera from SARS-CoV-2–negative animals.

Serum samples were collected on days 28 and 56, and control sera were obtained from unvaccinated animals. For the neutralization assay, two-fold serial dilutions of each serum sample were mixed with a constant amount of SARS-CoV-2-Δ19-D614G–pseudotyped lentivirus and incubated for 30 minutes at room temperature. The virus–serum mixtures were then added to 293T-hACE2 cells (BEI Resources, NIAID, NIH: Human Embryonic Kidney Cells Expressing Human ACE2, NR-52511) seeded the previous day at 3 × 10^4^ cells/well in black 96-well plates (PerkinElmer, #6005660). After 72 hours, fluorescence intensity was measured using a BioTek Synergy plate reader at room temperature (excitation: 485 nm; emission: 528 nm). The 50% neutralizing titer (ID_50_) was defined as the reciprocal of the serum dilution that reduced the number of infected cells by 50% relative to the virus-only control.

### 2.10 Statistical analysis

All values are expressed as mean ± standard deviation (SD) unless otherwise indicated. Statistical analyses were performed using GraphPad Prism. One-way ANOVA followed by Tukey’s multiple comparisons test was used to assess differences between groups. For RT-qPCR analysis, relative gene expression was calculated using the ΔΔCt method based on normalized Ct values, and statistical analyses were performed on ΔΔCt values. Standard deviations were calculated independently for each group based on biological replicates. The coefficient of variation (CV) was calculated for selected datasets as a descriptive measure of variability.

## 3 Results

### 3.1 Characterization and optimization of the automated intradermal delivery system

**Figure 1:**
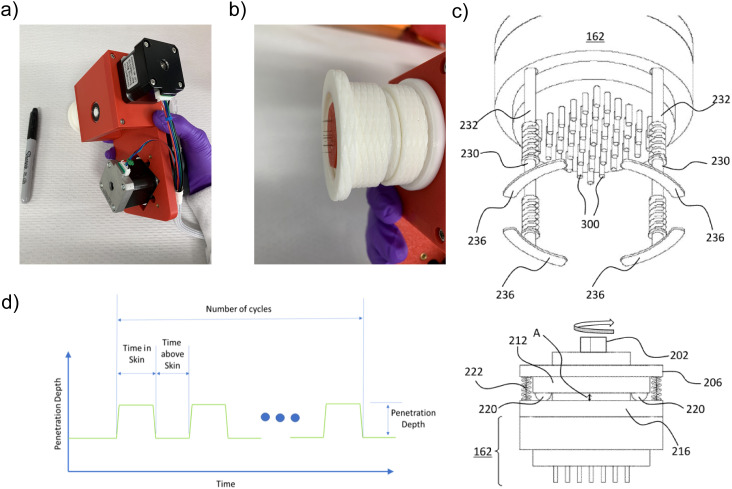
Device characteristics and operational parameters. **(a)** Photograph of the device held in one hand next to a marker to illustrate its size and ease of handling. **(b)** Close-up view of the adjustable needle array with defined spacing (pitch), enabling distributed intradermal injection. The 2.5 × 2.5 cm delivery area is defined by programmed rotational and translational movement of the microneedle array, enabling controlled spatial coverage through repeated micro-injections across a predefined dermal surface. **(c)** Schematic illustration of the needle-array operating sequence, with labels identifying the key components and programmed motion steps shown in the original patent drawing (US 20240075215 A1). **(d)** Schematic representation of the parameters defined in the program settings, where the y-axis indicates penetration depth and the x-axis represents time.

Building upon a previously reported intradermal tattoo-based injection device that enhanced the immunogenicity of plasmid DNA vaccines (15), we evaluated an updated prototype named InocuJect. The device was developed to enable controlled intradermal delivery while maintaining procedural safety and reliable vaccine administration. The automated ID device has a compact design that facilitates handling and stable skin contact over a 2.5 × 2.5 cm area (**Figure 1a**). Its rotating microneedle array, comprising 5–50 fine-gauge needles (**Figure 1b**), allows spatially distributed coverage of the injection zone. A schematic illustration of the needle-array configuration and motion sequence is provided in **Figure 1c**.

The device operates through mechanically and digitally programmable parameters controlling needle penetration depth, position, and timing. The microneedle array can penetrate the skin at adjustable depths from 0 to 4 mm, dwell within the dermis for 1 ms to 1 s, and retract at frequencies of 1–200 Hz. Each injection cycle delivers programmable micro-volumes (0–0.1 mL per pulse) over 1 ms to 1 s, allowing control over delivery kinetics (**Figure 1d**). Up to 1 mL of formulation can be administered within a few seconds. Automated rotation and translation of the needle array (up to 360° and 2 cm, respectively) distributes the vaccine across the target area.

### 3.2 Evaluation of the automated ID device using a DNA vaccine (pIDV-II-SARS-CoV-2)

**Figure 2:**
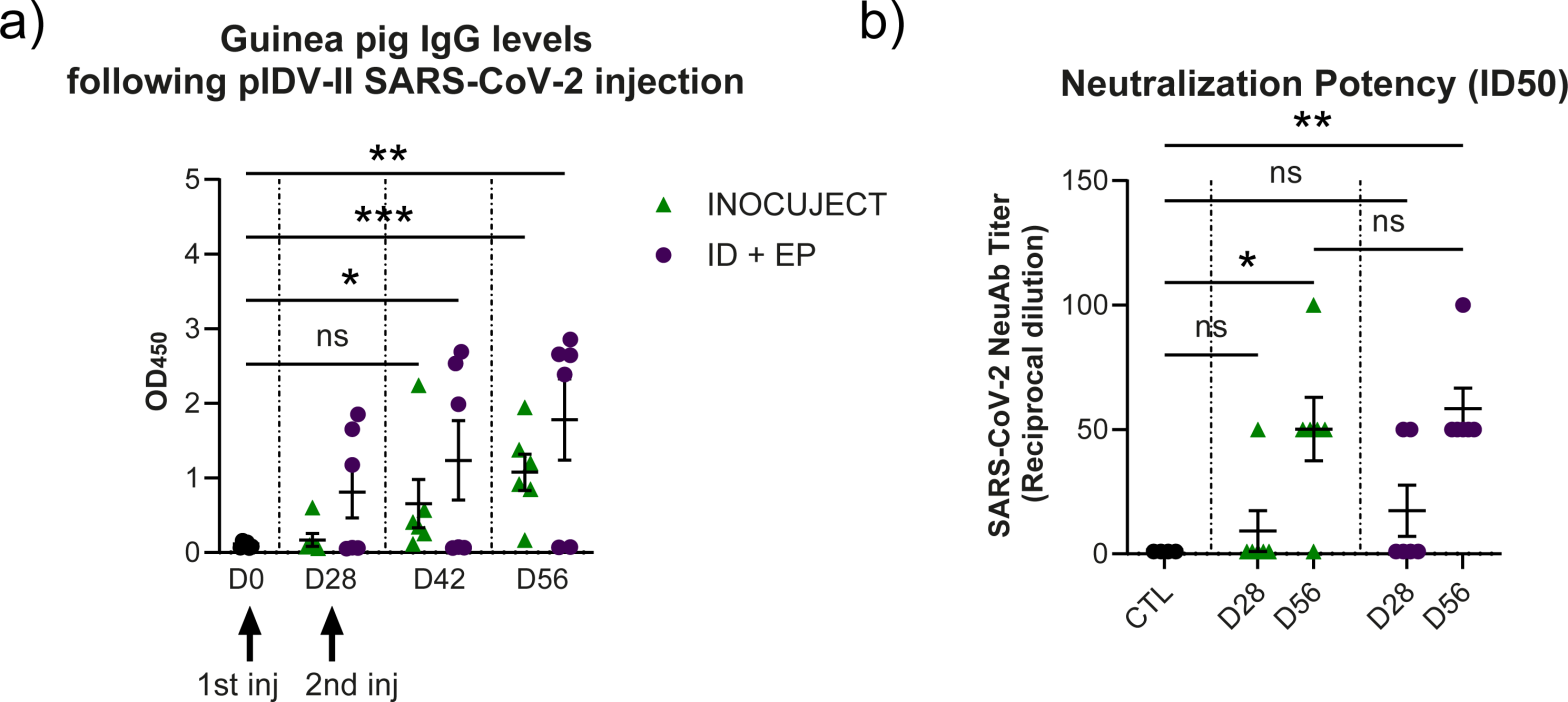
Humoral response and neutralizing antibody activity in guinea pigs vaccinated with the DNA vaccine pIDV-II-SARS-CoV-2. **(a)** SARS-CoV-2 spike-specific IgG levels were quantified by ELISA. Statistical analyses were performed using two-way ANOVA. Data represent mean ± SD (*p = 0.0343, **p = 0.0033, ***p = 0.0004; ns, not significant). Each group included six animals, and results are representative of at least two independent experiments, each performed with two technical replicates per sample. **(b)** Neutralizing activity was assessed using SARS-CoV-2 spike-pseudotyped lentiviruses with a luciferase reporter system. Control (CTL) sera from naïve guinea pigs and sera collected on days 28 and 56 post-vaccination were evaluated for their ability to achieve 50% neutralization (ID_50_). Data represent mean ± SEM of reciprocal dilutions from two independent experiments (n = 2), with *p = 0.0231 and **p = 0.0065. Data were analyzed using ordinary One-way ANOVA.

The automated ID device was evaluated using the same DNA vaccine construct (pIDV-II-SARS-CoV-2) previously tested with the original intradermal tattoo-based prototype (15). This vaccine has also demonstrated protective immunogenicity when delivered by electroporation (31), providing a relevant benchmark for intradermal delivery performance. The experimental design included prime and booster immunizations on Days 0 and 28 and serum collection on Days 0, 28, 42, and 56. Humoral responses were assessed by ELISA using the SARS-CoV-2 spike protein to measure IgG levels. As shown in **Figure 2a**, both groups showed a progressive increase in spike-specific IgG over the course of the study. After the first dose and booster, the ID + EP group exhibited a rapid and significant rise in OD_450_ values, reaching a mean of 1.235 ± 1.300 SD at Day 42 and 1.781 ± 1.332 SD at Day 56. In contrast, the InocuJect group displayed a slower response, with mean OD_450_ values of 0.656 ± 0.791 SD at Day 42 and 1.076 ± 0.594 SD at Day 56, representing a modest but significant increase compared to Day 0.

Although mean spike-specific IgG levels were higher in the ID + EP group, both delivery methods induced measurable antibody responses by Day 56. Of note, all animals in the group injected using the automated ID device demonstrated measurable antibody levels at the final time point, indicating a more uniform, although gradual, humoral response. These patterns are consistent with trends observed in commercial vaccine studies. Overall, the automated ID device elicited a slower but consistent antibody response appropriate for a DNA vaccine, whereas ID + EP induced a faster response but with incomplete seroconversion across animals.

Neutralizing antibody titers were evaluated using a SARS-CoV-2 spike–pseudotyped lentiviral fluorescent reporter system in HEK293 cells expressing the human ACE2 receptor. The reciprocal serum dilution required to neutralize 50% of viral entry is shown in **Figure 2b**. Both groups exhibited significant neutralization at day 56, with titers of 50.17 ± 31.31 SD for the InocuJect group and 58.33 ± 20.41 SD for the ID + EP group. Although the ID + EP group showed slightly higher mean titers, the difference between the two groups at day 56 was not statistically significant. Overall, both delivery systems effectively induced the production of neutralizing antibodies by day 56.

### 3.3 Humoral immune response induced by GARDASIL^®^9 following intradermal vaccination with the automated ID device

**Figure 3:**
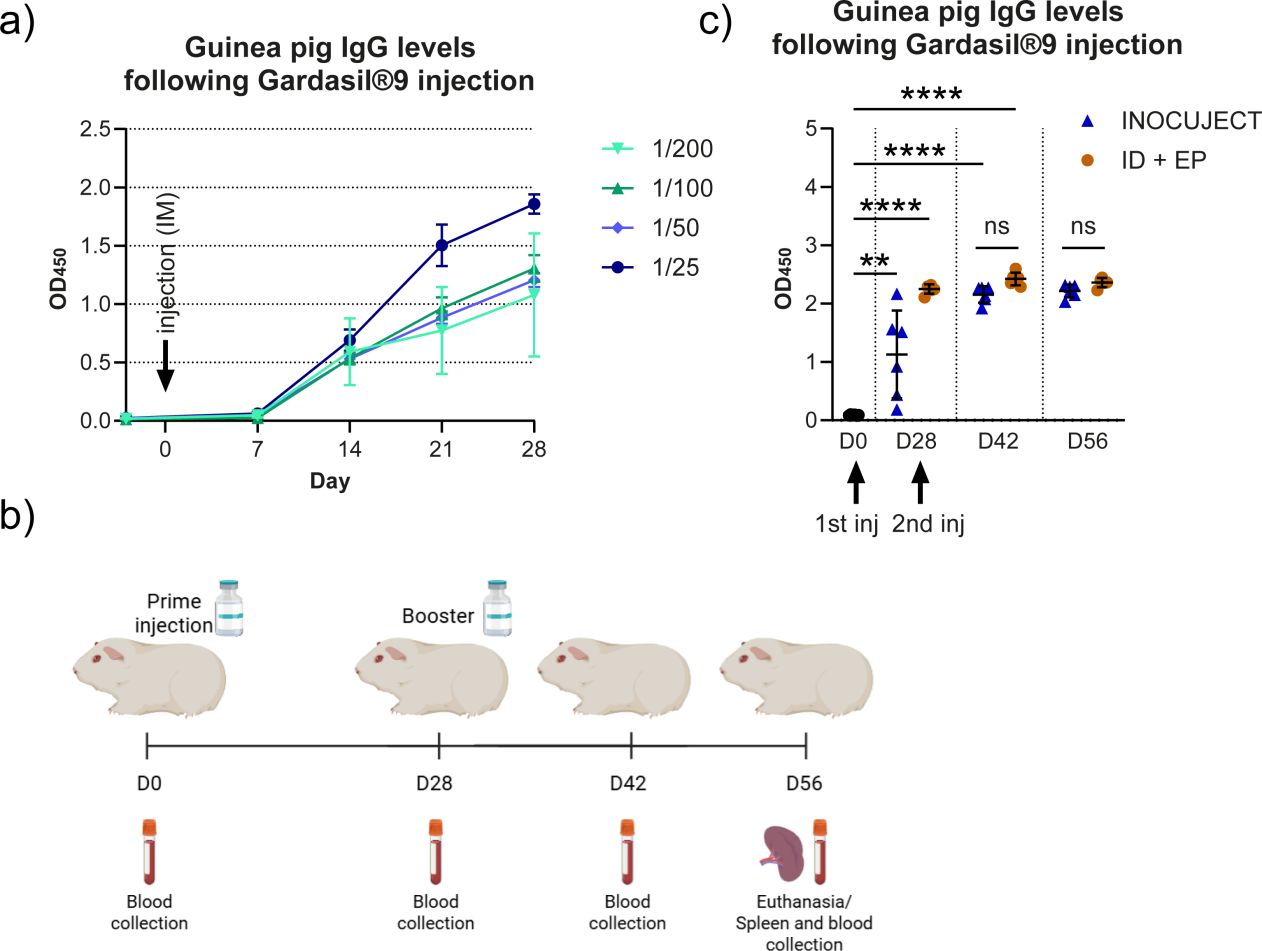
Evaluation of vaccine dose and humoral immune response to Gardasil^®^9 in guinea pigs. **(a)** ELISAs were performed using HPV16 L1 virus-like particles (VLPs) to quantify IgG levels based on optical density at 450 nm (OD_450_) and to evaluate four different doses of Gardasil^®^9. Data represent the mean ± SEM of three animals, each analyzed in duplicate. **(b)** Experimental design and timeline of the study showing vaccination, blood collection, and spleen harvest time points, comparing intradermal delivery using the automated ID device with the Mantoux method followed by electroporation (ID + EP) in guinea pigs. c) HPV16 L1-specific IgG levels were measured by ELISA. Statistical analyses were performed using a mixed-effects model to compare values with the day 0 (D0) control, and a one-way ANOVA to compare groups. Data represent the mean ± SD (**, p = 0.0018, ****, p < 0.0001; ns, not significant). Each group contained 5–6 animals, and results are representative of at least two independent experiments with two technical replicates per sample.

To determine the optimal dose of the commercial vaccine GARDASIL^®^9 formulated for adult human use in guinea pigs, four different dilutions ranging from 1/200 to 1/25 of the human dose were evaluated using the standard intramuscular route of administration. Sera from all four groups were collected prior to immunization and at multiple time points up to four weeks post-injection to assess the levels of antigen-specific immunoglobulin G (IgG) against the HPV16 L1 protein. As shown in **Figure 3a**, the immune response increased proportionally with the vaccine dose. To prevent signal saturation and ensure optimal immunogenicity in guinea pigs, the 1/100 dilution was selected for subsequent experiments.

Guinea pigs were then immunized with the vaccine using either the automated ID device (n = 6) or the intradermal Mantoux injection followed by electroporation, ID + EP, (n = 6, reduced to n = 5 by Day 56 due to one non–study-related death). Animals received the first dose on Day 0 and second dose on Day 28. For serum isolation, blood samples were collected at Days 0, 28, 42, and 56 after the first dose, while spleens were harvested at Day 56 for splenocyte isolation (**Figure 3b**). Of note, total volumes differed (0.5 mL automated ID device vs. 0.1 mL Mantoux) due to delivery design, with equivalent antigen doses (as detailed in Methods).

The humoral response was assessed by IgG ELISA using HPV16 L1 proteins, with serum collected before immunization and on Days 0, 28, 42, and 56 (**Figure 3c**). Following the prime injection at Day 28, IgG levels increased significantly in both groups compared to Day 0, with a mean OD_450_ of 1.13 ± 0.75 SD for the InocuJect group (p = 0.0018) and 2.251 ± 0.079 SD for the ID + EP group (p < 0.0001). While the group injected with the automated ID device showed a slower initial response, both groups reached similarly high IgG levels after the booster at Day 42 (2.155 ± 0.142 SD and 2.422 ± 0.107 SD for InocuJect and ID + EP, respectively). No significant differences in IgG levels were observed between the groups at Days 42 and 56, indicating that both delivery methods ultimately elicited comparable humoral responses.

### 3.4 Cellular immune response induced by GARDASIL^®^9

**Figure 4:**
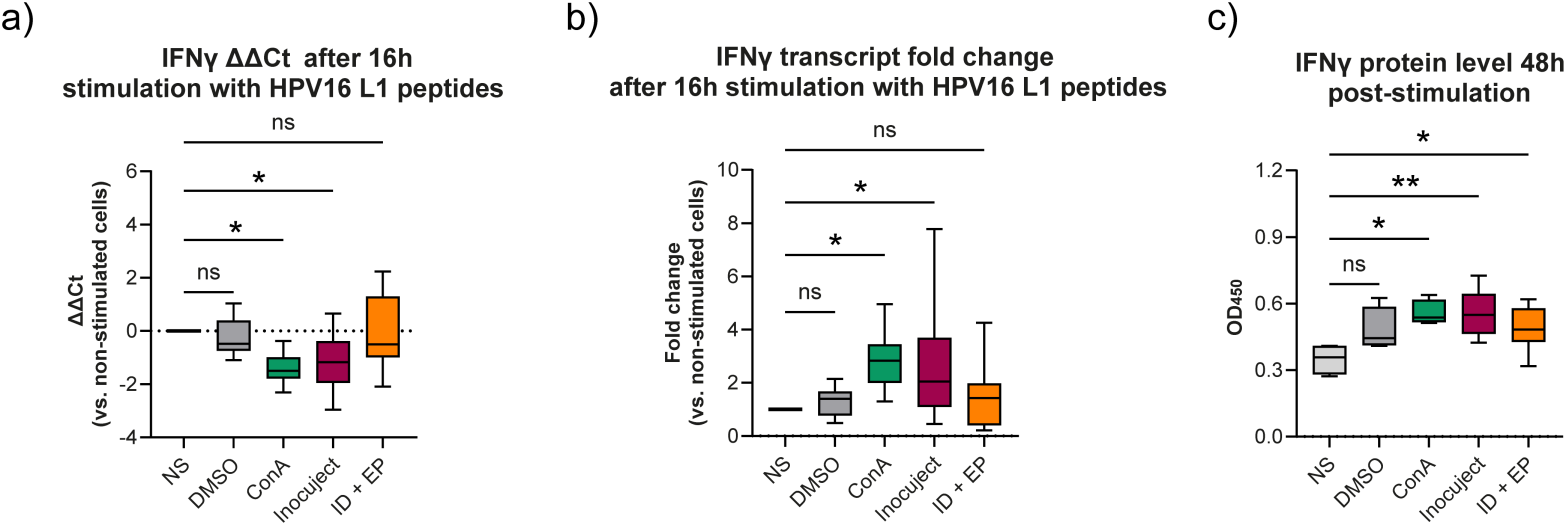
Cellular immune response and interferon gamma (IFNγ) levels in guinea pigs vaccinated with Gardasil^®^9. **(a)** RNA was extracted from guinea pig splenocytes 16 h after stimulation with HPV16 L1-specific peptides. Non-stimulated (NS) cells served as the baseline control, while DMSO and concanavalin A (ConA) were used as vehicle and positive controls, respectively. RT-qPCR targeting IFNγ, normalized to RnaseP as a housekeeping gene, was performed to evaluate relative RNA expression. ΔΔCt values were calculated from at least three animals across three independent experiments. Data are presented as box-and-whisker plots showing minimum to maximum values; because whiskers represent the full range of data, they may overlap between groups even when differences are statistically significant. Statistical significance was determined using ΔΔCt values derived from paired stimulated versus non-stimulated samples for each animal and analyzed by one-way ANOVA (NS vs. ConA, * p = 0.0172; NS vs. Inocuject, * p = 0.0420). **(b)** Fold change in IFNγ expression was calculated from ΔΔCt values using the 2^(-ΔΔCt) method. **(c)** IFNγ protein levels were measured by sandwich ELISA 48 h after splenocyte stimulation under the same conditions. Data are representative of at least two independent experiments with a minimum of three animals per group, presented as box-and-whisker plots (min to max), and analyzed using a one-way ANOVA followed by a Dunnett’s multiple comparison test (NS vs. ConA, * p = 0.0116; NS vs. InocuJect, ** p = 0.0016; NS vs. ID + EP, * p = 0.0434).

To evaluate the antigen-specific cellular response, interferon gamma (IFNγ) expression was measured in splenocytes stimulated with a pool of peptides specific to the HPV16 L1 protein. RNA was extracted 16 hours post-stimulation, and IFNγ levels were quantified by RT-qPCR using guinea pig-specific primer-probes, with RnaseP serving as the housekeeping gene. Raw cycle threshold (Ct) values and ΔΔCt values (IFNγ normalized to RnaseP and to non-stimulated (NS) cells) are presented in **Figure 4a**. For visualization, fold-change values were calculated and plotted in **Figure 4b**, while statistical analyses were performed using the ΔΔCt data.

Vehicle control (DMSO) had minimal effect (mean ΔΔCt = −0.2513 ± 0.712; fold-change = 1.31). Cells stimulated with Concanavalin A (ConA) showed a significant increase (* p = 0.0172) in IFNγ expression (mean ΔΔCt = −1.418 ± 0.586 SD; fold-change = 2.86). Similarly, splenocytes from animals vaccinated using the automated ID device and stimulated with HPV16 L1 peptides exhibited a significant increase (* p = 0.042) in IFN-γ expression (mean ΔΔCt = −1.104 ± 1.006 SD; fold-change = 2.53). In contrast, cells from the ID + EP group showed no significant difference following peptide stimulation (mean ΔΔCt = −0.0247 ± 1.354 SD; fold-change = 1.44).

The level of IFNγ protein was assessed using a sandwich ELISA specific for guinea pig interferon. As shown in **Figure 4c**, vehicle control (DMSO) did not significantly increase IFNγ protein levels, whereas ConA and both vaccination groups showed significant increases compared to non-stimulated (NS) cells. The mean OD_450_ values were 0.4805 ± 0.099 SD (CV = 20.57%) for DMSO, 0.5565 ± 0.057 SD (CV = 10.18%) for ConA (* p = 0.0116), 0.5595 ± 0.102 SD (CV = 18.17%) for InocuJect (** p = 0.0016), and 0.4885 ± 0.096 SD (CV = 19.72%) for ID + EP (* p = 0.0434), consistent with the IFN-γ RNA expression results. Variability assessed using the coefficient of variation (CV) indicated moderate dispersion across biological replicates. Overall, the automated ID device was associated with increased IFN-γ responses in peptide-stimulated splenocytes, while humoral responses remained comparable between groups.

### 3.5 Local tolerability and animal well-being

**Figure 5:**
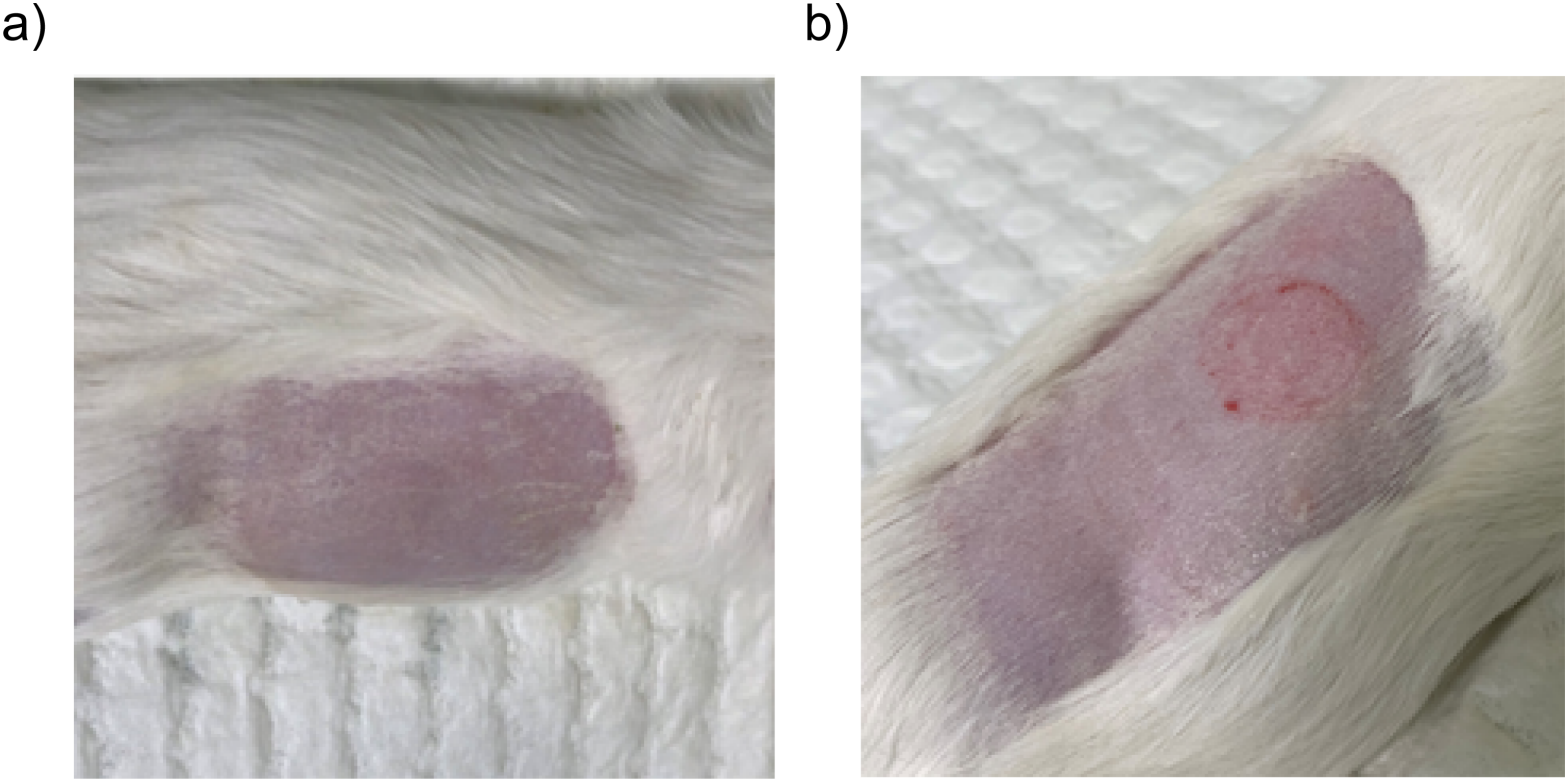
Representative images of the injection site following administration using the automated ID device. **(a)** Guinea pig after shaving and prior to vaccination. **(b)** Guinea pig following vaccination, showing the highest level of local irritation observed during the study period.

In guinea pigs, administration with the automated ID device resulted in none or mild and transient local irritation (**Figures 5a, b**, before and after injection, respectively), as illustrated by representative skin image of the highest level of local irritation observed during the study period. No ulceration or adverse local reactions were observed in group vaccinated with either the automated ID device or the electroporation device. All animals maintained normal grooming behavior, feeding patterns, and activity levels throughout the study period. These observations are consistent with previously reported tolerability profiles of microneedle-based and electroporation-enhanced intradermal vaccine delivery systems (32, 33).

## 4 Discussion

In this study, we evaluated the immunogenic performance of a programmable intradermal microneedle-array device in a guinea pig model using two distinct vaccine platforms: the DNA-based pIDV-II-SARS-CoV-2 and the VLP-based GARDASIL^®^9. Each vaccine served as an independent model to evaluate how the delivery method influences immunogenicity, rather than for direct comparison between the vaccine platforms. Across both models, the automated ID device elicited robust humoral responses comparable to those obtained with conventional ID Mantoux injections followed by electroporation, while being associated with stronger antigen-specific cellular responses as reflected by increased IFN-γ expression.

For the DNA vaccine, both delivery approaches induced detectable spike-specific IgG and neutralizing antibody response by day 56, with no statistically significant difference in neutralizing titers between groups. While the ID + EP group showed a more rapid increase in IgG antibody levels, the automated ID device resulted in a more gradual response profile, with all animals reaching measurable antibody levels at the final time point. These observations suggest that, although electroporation may accelerate immune activation, spatially distributed intradermal delivery can achieve comparable humoral outcomes over time. It is worth noting that the imperfect alignment between total IgG and neutralization activity aligns with prior SARS-CoV-2 studies (34), where low-level antibodies can retain functional activity at lower assay dilutions. These observations reflect the slower kinetics of DNA vaccines, in which germinal-center reactions and affinity maturation continue for several weeks post-boost (35).

Similarly, in the context of GARDASIL^®^9 vaccine, both delivery methods generated comparable antigen-specific IgG responses with no significant differences in final titers, whereas ID + EP accelerated early antibody induction, consistent with prior reports that electroporation enhances immune activation for DNA, protein, and VLP-based vaccines. This enhancement is attributed to increased tissue permeability, transient local inflammation, and improved recruitment and activation of antigen-presenting cells (APCs) (16, 36, 37). In contrast, the automated ID device is designed to distribute antigen across a broader 2.5 × 2.5 cm dermal field, resulting in spatially distributed multi-site deposition. This delivery pattern may increase the likelihood of interaction with skin-resident antigen-presenting cells, such as Langerhans cells and dermal dendritic cells, as suggested by previous intradermal and microneedle-based delivery studies (10, 38)). Despite differences in early kinetics, both methods ultimately generated comparable final antibody levels, demonstrating that the automated ID device effectively supports dermal delivery of VLP-based vaccines.

A difference between delivery methods was observed in cellular immunity. In the GARDASIL^®^9 model, the automated ID device elicited robust antigen-specific IFN-γ responses detectable by both RT-qPCR and protein ELISA, whereas ID + EP induced lower or non-significant responses. These findings indicate that the automated ID device supports antigen-specific cellular responses following VLP-based vaccines. The broader dermal coverage achieved by the automated ID device may engage a larger population of antigen-presenting cells (APCs), including Langerhans cells and dermal dendritic cells, which could contribute to the strength of cellular responses. Robust T-cell responses, particularly IFN-γ production, are critical for antiviral immunity and long-term protection (39, 40). The consistency observed across animals, as supported by coefficient of variation analysis, further underscores the ability of the device to induce measurable cellular immune responses.

Although the automated ID device and ID + EP use different injection volumes (0.5 mL vs. 0.1 mL), the antigen dose was identical in both groups. Prior studies indicate that intradermal immune activation correlates with antigen quantity rather than injection volume (41, 42), confirming that observed immunological differences reflect delivery characteristics rather than dosing disparities.

Certain aspects of the study design should be considered when interpreting these findings. The device was calibrated prior to each use to support controlled delivery; however, a detailed quantitative characterization of delivery precision was not performed and was beyond the scope of this study, which primarily focused on *in vivo* biological outcomes. In addition, while the programmed motion of the device defines a controlled application area, direct visualization of antigen distribution within the dermis was not performed and would be of interest in future studies to further elucidate spatial delivery patterns. Finally, the study was designed to compare the automated platform to an established enhanced intradermal delivery approach (ID + EP) and was not intended to separate the contribution of individual procedural components; accordingly, sham control or ID-only groups were not included. This study was limited to a single small-animal model and two vaccine platforms, and future work in larger models and across additional formulations will be required to further define translational potential.

Beyond vaccination, programmable intradermal delivery platforms are increasingly being explored for applications including allergen immunotherapy, cancer therapy, and cell-based dermal interventions (43, 44). A system capable of controlled deposition across a defined skin area may provide advantages in settings where localized dermal exposure is important.

Taken together, these results demonstrate that a programmable intradermal needle-array device can achieve robust humoral immune responses comparable to electroporation-based delivery across distinct vaccine platforms, while supporting enhanced antigen-specific cellular responses. This approach offers a promising alternative to more complex delivery methods and supports further investigation into spatially distributed intradermal vaccination strategies.

## Data Availability

The original contributions presented in the study are included in the article/supplementary material. Further inquiries can be directed to the corresponding author.

## References

[1] Chen X . Emerging adjuvants for intradermal vaccination. Int J Pharm. (2023) 632:122559. doi: 10.1016/j.ijpharm.2022.122559. PMID: 36586639 PMC9794530

[2] Lafouresse F Groom JR . A task force against local inflammation and cancer: Lymphocyte trafficking to and within the skin. Front Immunol. (2018) 9:2454. doi: 10.3389/fimmu.2018.02454. PMID: 30405637 PMC6207597

[3] Nguyen AV Soulika AM . The dynamics of the skin's immune system. Int J Mol Sci. (2019) 20. doi: 10.3390/ijms20081811. PMID: 31013709 PMC6515324

[4] Combadiere B Liard C . Transcutaneous and intradermal vaccination. Hum Vaccin. (2011) 7:811–27. doi: 10.4161/hv.7.8.16274. PMID: 21817854

[5] Kim YC Prausnitz MR . Enabling skin vaccination using new delivery technologies. Drug Delivery Transl Res. (2011) 1:7–12. doi: 10.1007/s13346-010-0005-z. PMID: 21799951 PMC3143039

[6] Sticchi L Alberti M Alicino C Crovari P . The intradermal vaccination: Past experiences and current perspectives. J Prev Med Hyg. (2010) 51:7–14. doi: 10.3390/ijms20081811 20853670

[7] Brooks JT Marks P Goldstein RH Walensky RP . Intradermal vaccination for monkeypox - Benefits for individual and public health. N Engl J Med. (2022) 387:1151–3. doi: 10.1056/nejmp2211311. PMID: 36044621

[8] Facciola A Visalli G Lagana A Di Pietro A . An overview of vaccine adjuvants: Current evidence and future perspectives. Vaccines (Basel). (2022) 10. doi: 10.3390/vaccines10050819 PMC9147349 35632575

[9] Donnelly RF Raj Singh TR Woolfson AD . Microneedle-based drug delivery systems: Microfabrication, drug delivery, and safety. Drug Delivery. (2010) 17:187–207. doi: 10.3109/10717541003667798. PMID: 20297904 PMC2906704

[10] Kim YC Park JH Prausnitz MR . Microneedles for drug and vaccine delivery. Adv Drug Delivery Rev. (2012) 64:1547–68. doi: 10.1016/j.addr.2012.04.005. PMID: 22575858 PMC3419303

[11] Prausnitz MR Langer R . Transdermal drug delivery. Nat Biotechnol. (2008) 26:1261–8. doi: 10.1038/nbt.1504. PMID: 18997767 PMC2700785

[12] Forster A Junger M . Opportunities and challenges for commercializing microarray patches for vaccination from a MAP developer's perspective. Hum Vaccin Immunother. (2022) 18:2050123. doi: 10.1080/21645515.2022.2050123. PMID: 35356872 PMC9196745

[13] Menon I Bagwe P Gomes KB Bajaj L Gala R Uddin MN . Microneedles: A new generation vaccine delivery system. Micromachines (Basel). (2021) 12. doi: 10.3390/mi12040435. PMID: 33919925 PMC8070939

[14] Suh H Shin J Kim YC . Microneedle patches for vaccine delivery. Clin Exp Vaccine Res. (2014) 3:42–9. doi: 10.7774/cevr.2014.3.1.42. PMID: 24427762 PMC3890449

[15] Gomez AM Babuadze GG Plourde-Campagna MA Azizi H Berger A Kozak R . A novel intradermal tattoo-based injection device enhances the immunogenicity of plasmid DNA vaccines. NPJ Vaccines. (2022) 7:172. doi: 10.1038/s41541-022-00581-y. PMID: 36543794 PMC9771775

[16] Sardesai NY Weiner DB . Electroporation delivery of DNA vaccines: Prospects for success. Curr Opin Immunol. (2011) 23:421–9. doi: 10.1016/j.coi.2011.03.008. PMID: 21530212 PMC3109217

[17] van Drunen Littel-van den Hurk S Hannaman D . Electroporation for DNA immunization: Clinical application. Expert Rev Vaccines. (2010) 9:503–17. doi: 10.1586/erv.10.42. PMID: 20450325

[18] Fagone P Shedlock DJ Kemmerer S Rabussay D Weiner DB . Electroporation-mediated DNA vaccination. In: Kee ST Gehl J Lee EW , editors.Clinical aspects of electroporation. Springer New York, New York, NY (2011). p. 203–15.

[19] Lin F Shen X Kichaev G Mendoza JM Yang M Armendi P . Optimization of electroporation-enhanced intradermal delivery of DNA vaccine using a minimally invasive surface device. Hum Gene Ther Methods. (2012) 23:157–68. doi: 10.1089/hgtb.2011.209. PMID: 22794496 PMC4015073

[20] Roos AK Eriksson F Walters DC Pisa P King AD . Optimization of skin electroporation in mice to increase tolerability of DNA vaccine delivery to patients. Mol Ther. (2009) 17:1637–42. doi: 10.1038/mt.2009.120. PMID: 19532140 PMC2835273

[21] Hettinga J Carlisle R . Vaccination into the dermal compartment: Techniques, challenges, and prospects. Vaccines (Basel). (2020) 8. doi: 10.3390/vaccines8030534. PMID: 32947966 PMC7564253

[22] Verbist W Broeckhoven E Vanwersch P Shrirang Choudhari V Van Hileghem L Spasic D . Advancing intradermal vaccine delivery: Focus on hollow microneedles and skin models. Hum Vaccin Immunother. (2026) 22:2619235. doi: 10.1080/21645515.2026.2619235. PMID: 41614604 PMC12867454

[23] Levin Y Kochba E Hung I Kenney R . Intradermal vaccination using the novel microneedle device MicronJet600: Past, present, and future. Hum Vaccin Immunother. (2015) 11:991–7. doi: 10.1080/21645515.2015.1010871. PMID: 25745830 PMC4514308

[24] Nguyen HX . Beyond the needle: Innovative microneedle-based transdermal vaccination. Medicines (Basel). (2025) 12. doi: 10.3390/medicines12010004. PMID: 39982324 PMC11843882

[25] Edupuganti S S CDR Elizaga M Lu Y Han X Huang Y . Intramuscular and intradermal electroporation of HIV-1 PENNVAX-GP((R)) DNA vaccine and IL-12 is safe, tolerable, acceptable in healthy adults. Vaccines (Basel). (2020) 8. doi: 10.3390/vaccines8040741. PMID: 33297341 PMC7762306

[26] Canada M . GARDASIL 9 – product monograph. (2021).

[27] Einstein MH Baron M Levin MJ Chatterjee A Edwards RP Zepp F . Comparison of the immunogenicity and safety of Cervarix and Gardasil human papillomavirus (HPV) cervical cancer vaccines in healthy women aged 18–45 years. Hum Vaccin. (2009) 5:705–19. doi: 10.1016/s0140-6736(13)60022-7. PMID: 19684472

[28] Kawahara M Nakasone T Honda M . Dynamics of gamma interferon, interleukin-12 (IL-12), IL-10, and transforming growth factor beta mRNA expression in primary Mycobacterium bovis BCG infection in Guinea pigs measured by a real-time fluorogenic reverse transcription-PCR assay. Infect Immun. (2002) 70:6614–20. doi: 10.1128/iai.70.12.6614-6620.2002. PMID: 12438333 PMC132987

[29] Sarkadi J Jankovics M Fodor K Kis Z Takacs M Visontai I . High-level cellular and humoral immune responses in Guinea pigs immunized intradermally with a heat-inactivated varicella-zoster virus vaccine. Clin Vaccine Immunol. (2015) 22:570–7. doi: 10.1128/cvi.00773-14. PMID: 25787138 PMC4412949

[30] Ferkova S Froehlich U Nepveu-Traversy ME Murza A Azad T Grandbois M . Comparative Analysis of Cyclization Techniques in Stapled Peptides: Structural Insights into Protein-Protein Interactions in a SARS-CoV-2 Spike RBD/hACE2 Model System. Int J Mol Sci. (2023) 25(1). doi: 10.3390/ijms25010166. PMID: 38203338 PMC10778704

[31] Babuadze GG Fausther-Bovendo H deLaVega MA Lillie B Naghibosadat M Shahhosseini N . Two DNA vaccines protect against severe disease and pathology due to SARS-CoV-2 in Syrian hamsters. NPJ Vaccines. (2022) 7:49. doi: 10.1038/s41541-022-00461-5. PMID: 35474311 PMC9042934

[32] Hung IFN Yuen KY . Immunogenicity, safety and tolerability of intradermal influenza vaccines. Hum Vaccin Immunother. (2018) 14:565–70. doi: 10.1080/21645515.2017.1328332. PMID: 28604266 PMC5861844

[33] Diehl MC Lee JC Daniels SE Tebas P Khan AS Giffear M . Tolerability of intramuscular and intradermal delivery by CELLECTRA((R)) adaptive constant current electroporation device in healthy volunteers. Hum Vaccin Immunother. (2013) 9:2246–52. doi: 10.4161/hv.24702. PMID: 24051434 PMC3906411

[34] Higashimoto Y Kozawa K Miura H Kawamura Y Ihira M Hiramatsu H . Correlation between anti-S IgG and neutralizing antibody titers against three live SARS-CoV-2 variants in BNT162b2 vaccine recipients. Hum Vaccin Immunother. (2022) 18:2105611. doi: 10.1080/21645515.2022.2105611. PMID: 36094467 PMC9746447

[35] Yang H Zhou Y Cheng X Qiu C Wang S Xia Y . Safety, tolerability, and immunogenicity of a DNA vaccine (pGX9501) against SARS-CoV-2 in healthy volunteers: A single-center, randomized, double-blind, placebo-controlled, and dose-ranging phase I trial. Vaccines (Basel). (2025) 13. doi: 10.3390/vaccines13060573. PMID: 40573904 PMC12197376

[36] Mesin L Ersching J Victora GD . Germinal center B cell dynamics. Immunity. (2016) 45:471–82. doi: 10.1016/j.immuni.2016.09.001. PMID: 27653600 PMC5123673

[37] Low L Mander A McCann K Dearnaley D Tjelle T Mathiesen I . DNA vaccination with electroporation induces increased antibody responses in patients with prostate cancer. Hum Gene Ther. (2009) 20:1269–78. doi: 10.1089/hum.2009.067. PMID: 19619001

[38] Kupper TS Fuhlbrigge RC . Immune surveillance in the skin: Mechanisms and clinical consequences. Nat Rev Immunol. (2004) 4:211–22. doi: 10.1038/nri1310. PMID: 15039758 PMC7097017

[39] Kang S Brown HM Hwang S . Direct antiviral mechanisms of interferon-gamma. Immune Netw. (2018) 18:e33. doi: 10.4110/in.2018.18.e33. PMID: 30402328 PMC6215902

[40] Whitmire JK Benning N Whitton JL . Cutting edge: Early IFN-gamma signaling directly enhances primary antiviral CD4+ T cell responses. J Immunol. (2005) 175:5624–8. doi: 10.4049/jimmunol.175.9.5624. PMID: 16237051

[41] Egunsola O Clement F Taplin J Mastikhina L Li JW Lorenzetti DL . Immunogenicity and safety of reduced-dose intradermal vs intramuscular influenza vaccines: A systematic review and meta-analysis. JAMA Netw Open. (2021) 4:e2035693-e. doi: 10.1001/jamanetworkopen.2020.35693. PMID: 33560425 PMC7873776

[42] Chuaychoo B Kositanont U Niyomthong P Rittayamai N Srisuma S Rattanasaengloet K . Comparison of immunogenicity between intradermal and intramuscular injections of repeated annual identical influenza virus strains post-pandemic (2011-2012) in COPD patients. Hum Vaccin Immunother. (2020) 16:1371–9. doi: 10.1080/21645515.2019.1692559. PMID: 31770051 PMC7482887

[43] Lallow EO Busha KJ Park SH Atzampou M Jhumur NC Demiryurek Y . Molecular distribution in intradermal injection for transfer and delivery of therapeutics. Front Drug Delivery. (2023) 3:1095181. doi: 10.3389/fddev.2023.1095181. PMID: 40838056 PMC12363334

[44] Leoni G Lyness A Ginty P Schutte R Pillai G Sharma G . Preclinical development of an automated injection device for intradermal delivery of a cell-based therapy. Drug Delivery Transl Res. (2017) 7:695–708. doi: 10.1007/s13346-017-0418-z. PMID: 28812281 PMC5574955

